# Self-Heating Mould for Composite Manufacturing

**DOI:** 10.3390/polym13183074

**Published:** 2021-09-12

**Authors:** Andrii Kondratiev, Václav Píštěk, Svitlana Purhina, Maryna Shevtsova, Anna Fomina, Pavel Kučera

**Affiliations:** 1 Department of Building Technology and Construction Materials, O.M. Beketov National University of Urban Economy in Kharkiv, Marshal Bazhanov Str. 17, 61002 Kharkiv, Ukraine; andrii.kondratiev@kname.edu.ua; 2 Institute of Automotive Engineering, Brno University of Technology, Technická 2896/2, 616 69 Brno, Czech Republic; pistek.v@fme.vutbr.cz; 3 Department of Composite Structures and Aviation Materials, National Aerospace University “Kharkiv Aviation Institute”, Chkalova Str. 17, 61070 Kharkiv, Ukraine; s.purhina@khai.edu (S.P.); m.shevtsova@khai.edu (M.S.); 4 Department of Railway, Automobile Transport and Handling Machines, Institute of Transport and Logistics, Volodymyr Dahl East Ukrainian National University, Central Avenue 59a, 93400 Sewerodonetsk, Ukraine; anyta220885@gmail.com

**Keywords:** resistive element, internal heating, exothermic effect, heating system power, laying pitch, thermal blanket

## Abstract

The shipbuilding industry, engine manufacturing, aviation, rocket and space technology are promising fields of application for polymeric composite materials. Shape-generating moulding tools with internal heating are used for the creation of a more economically viable method of moulding of internally heated composite structures. The use of a fine-fibered resistive structure in the heated tools allows implementation of effective heating of the composite and elimination of the need for expensive and energy-intensive heating equipment. The aim of this paper was the reduction of energy consumption for internally heated moulding tools by choosing the optimal parameters for their resistive layer. A method for determination of the parameters of the moulding tool resistive layer was developed. This method allows calculation of the heating layer parameters and implementation of the specified time–temperature regime for moulding of the composite structure. It was shown that energy saving for the heated fiberglass shape-generating moulding tools was from 40 to 60%. It was found that the increase in the thickness of the moulded package of the polymeric composite material resulted not only in a higher supplied power for the heating system, but also in a complication of the method for system control, because of the growing exothermic effect of the binder curing reaction. For composite products based on Hysol EA 9396 binder, thicknesses more than 4 mm are critical, because it is not possible to cope with the self-heating effect only by cooling with ambient air already utilized at the twentieth minute of the moulding process. The influence of the physical and mechanical characteristics of the moulding tool material and stiffening ribs was analysed in terms of energy consumption and controllability of the heating system. Fiberglass shows the lowest energy consumption. Heating of the aluminium and steel moulding tools for the same purpose will require 20% and 45% more power, respectively. An increase in the number of stiffening ribs has a strong effect on the heat removal of the heating system. With a small number of aluminium ribs it is not possible to maintain the specified temperature–time regime for a fiberglass moulded package of 5 mm thick with the use of the equipment. However, when the number of stiffeners is increased to 10, the exothermic effect of the reaction becomes smoother and then the heating equipment can cope with the task. An experimental prototype of heating equipment of moulding tools for the manufacturing of structures of polymeric composite materials, as well as a flexible thermal blanket for repair of non-separable structures, were developed. The results can be the basis for a new method of optimal design of parameters of moulding tool structure at minimal heat removal to the environment.

## 1. Introduction

At the present time, composite materials based on ultrathin carbon, glass, organic and other types of fibres in combination with polymeric binders are widely used in various branches of technology [1,2,3,4], such as general construction, bridge engineering, road infrastructure, transport, agricultural machinery, the power sector, biomedicine, and petro-chemistry [5,6,7,8,9,10]. The shipbuilding industry, engine manufacturing, aviation, rocket and space technology are the most promising fields of application of polymeric composite materials [11,12]. High specific strength and stiffness, as well as a number of other unique properties of the composites, which allow the implementation of special qualities in the structure, are in demand in these fields [13,14].

One of the most rational methods of repair of typical operational defects (cracks, penetration defects of skin, delaminations) in metal and composite panel constructions is the installation of patches made of polymeric composite materials [15,16]. The high physical and mechanical properties of the composites and the significant advantages of the adhesive joints over mechanical ones, determine the successful use of patches made of polymeric composite materials for the repair of both polymeric and metal structures of various applications [17,18,19]. Because the need for repair of structures in the future will continue to grow, increase in the efficiency of repair processes is no less important than improvement of the methods of designing of new structures [20,21,22].

Moulding of products of polymeric composite materials is performed, as a rule, with the use of heaters (furnaces or autoclaves) [23]. The efficiency of any heating method is determined by the ratio of produced and transferred heat, therefore the introduction of contact heaters in the manufacturing of composites is an urgent task [24]. It allows reduction of the time and costs for the production of composite structures. Now there are several areas in the development of heating equipment, which provide reduced energy consumption in the process of creation and reconditioning repair of composite structures. In order to implement new methods of composite structure moulding, binder compositions cured by ultraviolet radiation or another type of radiation have been developed, as well as heating complexes where the shaping surface of the tools is an integrated part [25]. However, such complexes are characterized, apart from high cost, by the great complexity of production, operation, and maintenance; therefore, they do not find wide industrial application.

For the creation of a more economically viable method for moulding of composite structures, heated moulding tools are developed, to which the heat is supplied by convective or contact heat transfer. The use of resistive blocks in the heated tools allows implementation of efficient heating of the polymeric composite materials without significant changes to the tool design [26]. The heating units can be manufactured on the basis of the fine-fibered resistive structure or plates/small cores. The main disadvantage of plates and units with a core is the gap occurring in the process of unit installation; the resulting temperature difference can be compensated only by a sufficient thickness of the shaping surface of the tool. Resistive blocks based on fine-fibered resistive structures are the only units, which allow implementation of the uniformity of the temperature field on the shaping surface, when the resistive blocks are installed with a gap equal to the pitch of the laying of the resistive thread [27].

Authors of the paper [28] propose a cure of the binder by applying an electric current to the carbon fibre composite part. It was shown that the electrical conductivity of carbon fibre allows individual fibres to act as heating elements. As a result, there is a large number of internal heating elements throughout the composite part. The degree of curing was compared to composites cured in the traditional way. The three-point bending test was used to determine the flexural strength and modulus of elasticity. The results showed that the proposed technology provided polymeric composite material parameters on a level with autoclave production.

A method of direct resistive heating of the composite workpiece for curing was proposed in [29]. The heat problem was solved numerically by the finite element method. In this case, the heat conduction equation and kinetics of the binder curing reaction in the composite workpiece were modelled. Since the resistivity of materials is temperature dependent, a system with nonlinear relationship was developed. Comparison of the obtained results with experimental data showed satisfactory repeatability.

The paper [30] deals with the study of the method of self-resistive electric heating for the rapid moulding of parts of carbon fibre reinforced plastic. For the generation of heat and direct curing of the binder, the use of the electric current passing through the carbon fibre was proposed. A self-resistive tool with an automatic temperature controller was developed. The paper presents the results of experiments on the moulding of composite parts with the use of the developed equipment. The degree of curing of various specimens was characterized, and their cross-sectional geometry and porosity evaluated. On the basis of the experimental results, the behaviour of the heat transfer of various processes was analysed.

To reduce the autoclave costs, the use of a self-heating composite mould made of carbon fibre composite was proposed in [31]. It was suggested to use the reinforced carbon fibre composite shaping surface as the heating elements. The paper shows that due to the very low coefficient of thermal expansion of carbon fibre composite, this material is an excellent choice for the manufacturing of such moulds. The predicted uniformity of the temperature field was confirmed experimentally using thermocouples and an infrared camera.

A self-heating composite tool with built-in resistive layer was developed in [32]. It was shown that this layer evenly distributes the heat in close proximity to the surfaces of the part while ensuring a high level of mechanical characteristics of the polymeric composite materials. Finite element modelling of heat transfer confirmed that the tool configuration and selected heating elements provide sufficient heat uniformity to achieve the desired binder curing. Nevertheless, the paper indicates the necessity to solve the problem of choosing the optimal position of the resistive layer, as well as further testing to verify and calibrate the system to obtain the the optimal degree of binder curing.

The method for calculation of the heated tools providing rigidity and service life, with the heating layer based on the resistive blocks, was proposed in [33]. The parameters of the heating system (material of the heating elements, their geometry, and material of the insulating system) and the diagram of its connection were determined from the condition of securing the required heating mode. The heating was controlled by changing the current strength during the process, determined from the solution of the heat conduction problem, taking into account the exothermic effect of the curing reaction and conditions of heat transfer on the surface.

The thermodynamic model of unsteady heat transfer during moulding of the polymeric composite material in heated tools was developed in [34]. The model allowed the temperature distribution over the thickness of the system under study to be obtained, the influence of the exothermic effect of the binder curing reaction to be evaluated, and the required power of the heating system to be determined. The disadvantage of this paper is the assumption that the model does not take into account the ribbing of the lower surface of the moulding tools. 

As shown from the review and analysis of the problem, the possibility of out-of-autoclave production of the composite parts with the use of moulding tools with internal heating has now been substantiated. It eliminates the need for the expensive and energy-intensive heating equipment. For the improvement of the preproduction activity through a reasonable choice of the design of the moulding tools, it is necessary to develop an integrated solution, which allows reducing costs and shortening the production cycle. Therefore, the aim of the paper was to reduce energy consumption for internally heated moulding tools by choosing the optimal parameters of the resistive layer. 

To achieve this purpose, it was necessary to solve the tasks below:

1. Development of the method for determination of the parameters of the resistive layer of moulding tools, which would allow the heating layer parameters to be calculated and the specified time-temperature regime for moulding of the composite structures to be implemented;
2. Construction of a thermodynamic model of unsteady heat transfer for the system “shaping the surface-polymeric composite material”;
3. Development of an experimental prototype of heating equipment for moulding tools for the manufacture of structures of polymeric composite materials.

## 2. Materials and Methods

The analytical solution of tasks was carried out using theoretical studies in the field of thermodynamics, the theory of thermoelasticity of laminated plates, electrical engineering, as well as experimental research under laboratory conditions with the use of standard equipment, instruments, or devices. 

Numerical validation of the developed method was carried out on the example of moulding equipment for the formation of package based on fiberglass and Hysol EA 9396 (Producer: Henkel AG & Co. KGaA) binder with the resistive element made of carbon.

The experimental prototypes of the heating equipment of the moulding tools and the flexible thermal blanket were made from fiberglass with the use of epoxy low-temperature binder with the resistive element made of carbon.

The distribution of temperature on the surface of the heated shape-generating moulding tools was obtained by means of Thermal Imager Fluke Ti20 (Fluke Europe B.V., P.O. Box 1186, 5602 BD Eindhoven, The Netherlands).

## 3. Theoretical Background

**Figure 1:**
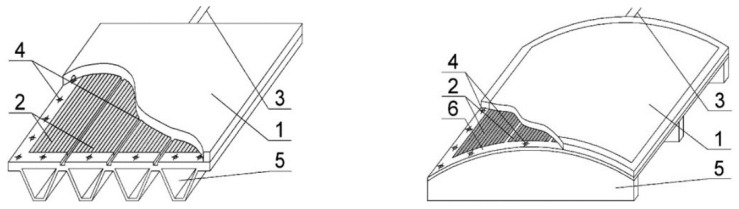
Moulding tool with the resistive layer, 1—shaping surface, 2—resistive blocks, 3—current-carrying wires, 4—mounting position, 5—frame, 6—resistive layer.

A moulding tool is a shaping surface, which is supported by a frame (Figure 1).

Determination of the temperature pattern of such a system is based on solving the heat conduction problem for a multilayer wall, as well as a ribbed surface [33]. To assess the impact of the system under study on environmental parameters, or vice versa, a method of thermodynamic research is used. It consists in tracking the thermodynamic changes when the system is isolated from the environment [34].

**Figure 2:**
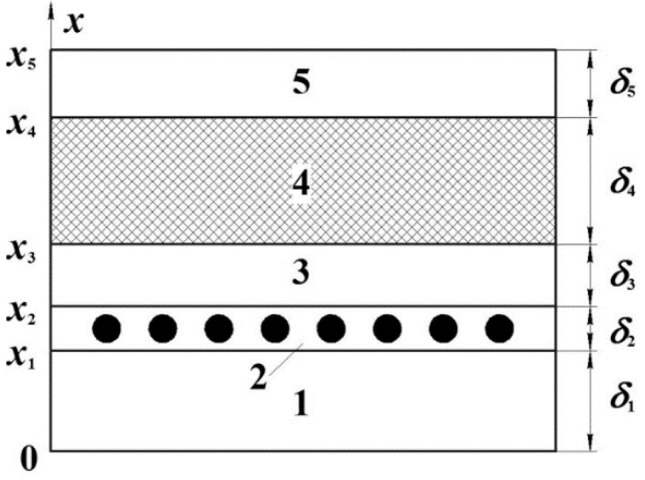
Thermodynamic system: 1—lower layer of the thermal fixture, 2—resistive layer with internal heat sources, 3—upper layer of the thermal fixture, 4—moulded package of polymeric composite material, 5—auxiliary layer.

We used the mathematical model of unsteady heat transfer [34] for the flat stiffened moulding tool with an area of working zone of *a* × *b* and the moulded package of polymeric composite material laid on it. The stiffening ribs of the moulding tool were made of metal and are located perpendicular to the larger side *a*; the shaping surface was made of the composite. The system under study consisted of five layers: the lower layer of the thermal fixture, the resistive layer with internal heat sources, the upper layer of the thermal fixture, the moulded package of polymeric composite material, and the auxiliary layer (Figure 2).

The system of discretely located heating elements was replaced by a homogeneous layer, the thickness of which was determined from the condition of equality of the power of the internal heat sources. The electric energy supplied to the moulding tool is consumed in maintaining the given rate of heating of the product and for the compensation of heat losses resulting from convective heat transfer from the surfaces of the heating system.

When constructing the thermodynamic model of the system under study, we made the assumptions below [34]:

1. A uniform temperature distribution over the surface of the moulding tool; neglecting heat exchange from the ends;
2. The main radii of curvature of the shaping surface of the tool are much greater than its thickness, so the effect of curvature on the temperature distribution can be neglected;
3. The thermophysical properties of the material are constant within each layer and do not depend on the temperature;
4. There is a perfect contact at the boundaries of the layers of the heating system.

The assumptions made allow the use of the heat conduction equations of a plane wall to solve the problem; they are written separately for each layer of the system:

(1) $$c^{\left(1\right)}\rho^{\left(1\right)}\frac{\partial T^{\left(1\right)}}{\partial t}=\lambda^{\left(1\right)}\frac{\partial^{2}T^{\left(1\right)}}{\partial^{2}x};\;c^{\left(2\right)}\rho^{\left(2\right)}\frac{\partial T^{\left(2\right)}}{\partial t}=\lambda^{\left(2\right)}\frac{\partial^{2}T^{\left(2\right)}}{\partial^{2}x}+q_{e};\;c^{\left(3\right)}\rho^{\left(3\right)}\frac{\partial T^{\left(3\right)}}{\partial t}=\lambda^{\left(3\right)}\frac{\partial^{2}T^{\left(3\right)}}{\partial^{2}x};\;c^{\left(4\right)}\rho^{\left(4\right)}\frac{\partial T^{\left(4\right)}}{\partial t}=\lambda^{\left(4\right)}\frac{\partial^{2}T^{\left(4\right)}}{\partial^{2}x}+q_{r};\;c^{\left(5\right)}\rho^{\left(5\right)}\frac{\partial T^{\left(5\right)}}{\partial t}=\lambda^{\left(5\right)}\frac{\partial^{2}T^{\left(5\right)}}{\partial^{2}x},$$

where $c^{\left(k\right)},\rho^{\left(k\right)},\lambda^{\left(k\right)}$—respectively, specific heat, density and heat conduction coefficient of the k-th layer; $T^{\left(k\right)}$—temperature in the *k*-th layer as a function of the coordinate *x* and time *t*; $q_{e}$—specific heat flux created by the resistive layer; $q_{r}$—specific heat flux of the exothermic effect of the binder curing reaction.

In the process of solving the heat conduction problems with the internal heat sources, the conditions of heat exchange on the outer surfaces are of great importance, since they significantly affect the temperature distribution over the wall thickness. If a known temperature is maintained on both surfaces of the wall (boundary conditions of the first kind), it is equivalent to the fact that several heat fluxes are applied to the surfaces, which are necessary for maintaining this temperature. When simulating the real heating systems with the use of boundary conditions of the first kind, it is necessary to be sure that the required heat fluxes can be created by means of the existing equipment. When the heated moulding tools are used, it should be noted that cooling of the package of the polymeric composite material is carried out only due to convective heat exchange of the outer surface of the auxiliary layers and the lower surface of the moulding tool. It can be assumed that, with the significant heat effect of the curing reaction, the specified temperature regime may not be implemented at full scope, since cooling due to convective heat transfer only, will not be enough to align the temperature curve at the moment of intense overheating of the structure. In addition, it is necessary to consider the one-way supply of heat from the tool side in the system under study; therefore, it does not seem possible to maintain the given temperature on both surfaces of the polymeric composite material package. Thus, to solve the problem we used boundary conditions of the third kind, where the convective heat transfer is set on the outer surface of the auxiliary layers and the lower surface of the moulding tool. With the use of the above setting, it was possible to assess the feasibility of the selected temperature regime using the available equipment. Therefore, on the outer surfaces of the wall at $x=x_{5}$ we write the boundary conditions of the convective heat transfer as follows:

(2) $$-\lambda^{\left(5\right)}\frac{\partial T^{\left(5\right)}}{\partial x}=\alpha \left(T_{к}-T^{\left(5\right)}\right),$$

and conditions of equality of heat fluxes are written for the contact surfaces of the layers:

(3) $$x=x_{1}:T^{\left(1\right)}=T^{\left(2\right)};\lambda^{\left(1\right)}\frac{\partial T^{\left(1\right)}}{\partial x}=\lambda^{\left(2\right)}\frac{\partial T^{\left(2\right)}}{\partial x};\;x=x_{2}:T^{\left(2\right)}=T^{\left(3\right)};\lambda^{\left(2\right)}\frac{\partial T^{\left(2\right)}}{\partial x}=\lambda^{\left(3\right)}\frac{\partial T^{\left(3\right)}}{\partial x};\;x=x_{2}:T^{\left(3\right)}=T^{\left(4\right)};\lambda^{\left(2\right)}\frac{\partial T^{\left(3\right)}}{\partial x}=\lambda^{\left(4\right)}\frac{\partial T^{\left(4\right)}}{\partial x};\;x=x_{4}:T^{\left(4\right)}=T^{\left(5\right)};\lambda^{\left(4\right)}\frac{\partial T^{\left(4\right)}}{\partial x}=\lambda^{\left(5\right)}\frac{\partial T^{\left(5\right)}}{\partial x};$$

while on the lower surface of the moulding tool at x=0 the boundary conditions are written, taking into account the ribbing (not previously taken into account in [29]):

(4) $$\lambda^{\left(1\right)}\frac{\partial T^{\left(1\right)}}{\partial x}=\alpha \left(T_{к}-T^{\left(1\right)}\right)+\frac{T^{\left(1\right)}}{2}\left(\frac{\alpha P_{1}}{f_{1}m_{1}}\cosh\left(m_{1}L_{1}\right)+\frac{\alpha P_{2}}{f_{2}m_{2}}\cosh\left(m_{2}L_{2}\right)\right),$$

where *α*—coefficient of convection; *T*_к_—ambient temperature. Initial conditions at t=0 take the form: $T^{\left(1\right)}=T^{\left(2\right)}=T^{\left(3\right)}=T^{\left(4\right)}=T^{\left(5\right)}=T_{к}$.

The power of the internal heat sources caused by the exothermic effect of the binder curing reaction are written as follows

(5) $$q=\Delta H\left(1-\theta \right)\rho_{b}\frac{d\eta }{dt},$$

where ΔH—amount of heat released with the exothermic effect by a kilogram of the binder; *θ*—volumetric content of reinforcing filler in the composite; $\rho_{b}$—volume density of the binder; $\frac{d\eta }{dt}$—rate of chemical reaction or rate of curing; η—degree of curing; *t—* time.

Since the reaction rate depends on temperature, the system of heat conduction Equation (1) has to be solved together with the kinetic equation under the initial conditions (t=0)$\eta =\eta_{0}$, where $\eta_{0}$—initial degree of the binder curing.

**Figure 3:**
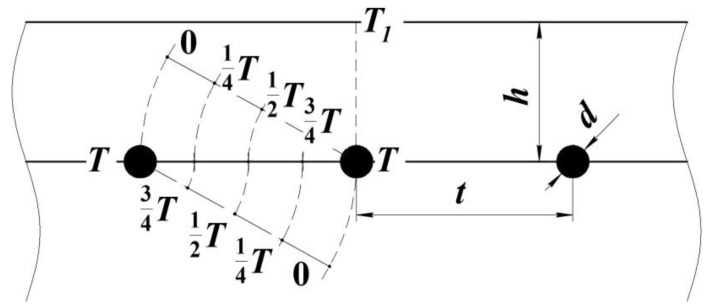
Shaping surface with internal heat sources.

The main characteristic of the resistive layer is the creation of a uniform temperature distribution over the shaping surface of the tool [30,31]. To satisfy this requirement, we determined the depth of the resistive layer deepening *h*, as well as the pitch of laying the heating elements *t* (Figure 3) for the moulding tool of power *N*.

The specific nature of the resistive structure introduces some differences in the heating system parameter calculation method [33,34].

Let us consider the temperature distribution from two heat sources of the resistive element based on a metal wire or carbon thread. As can be seen in Figure 3, the value of the laying pitch should be provided so that half of it corresponds to half of the temperature from the heating wire. Then, with the step-by-step laying of the next heating elements the temperature field will be even.

Using the equality of the heat flux $Q_{v}$ released by heat sources and the heat flux *Q*, passing through the distance of the laying pitch, as well as correspondence of the power of sources and the flow of released energy

(6) $$Q=Q_{v}=N$$

we obtain the following transformation of Fourier’s law:

(7) $$\frac{2\lambda }{t}F\left(T-\frac{T}{2}\right)=N$$

where F=2πrL—heating area, *r*—radius of heating wire with the length of *L*.

However, when choosing the diameter and length of the thread, it is important to consider two factors: the material of the elements of the internal sources and the heating surface area. For each material of the heating wire with diameter *d*, the known values are the maximum breakdown current *I_max_* and the resistance per unit length *ρ*, determined by the formula:

(8) $$\rho =\frac{R}{L},$$

where *R*—resistance of the material of the heating wire with the length of *L*.

Using the dependence for the power of current sources

(9) $$N=I^{2}R=IU=\frac{U^{2}}{R},$$

we determine the required resistance value

(10) $$R=\frac{N}{l_{max}^{2}}.$$

Based on the resistance value, the laying pitch is calculated as follows

(11) $$t=\frac{2\lambda T\pi rL}{N}=\frac{2\lambda T\pi r}{l_{max}^{2}\rho }.$$

Half of the laying pitch value determines the value of the deepening *h*. Therefore, parameters of the heating layer *t* and *h* for a specific conductor with the length of *L* are determined based on the required power of the system. However, in order to provide the required working area of heating, the length *L* of the conductor is not always sufficient. Therefore, at this stage of the calculation it is possible to change the initial parameters of the resistive element and recalculate the heating system parameters. As an alternative, it is possible to switch to a parallel wiring diagram. Let us give an algorithm which allows the required number of resistive blocks to be obtained forming a resistive layer of specified dimensions *a* × *b*. 

The area for laying of heating elements is determined by the formula

(12) $$F_{1}=b\left(n-1\right)t,$$

where *n*—number of pitches of laying of the heating elements, is determined by the formula:

(13) $$n=\frac{L+t}{b+t}.$$

**Figure 4:**
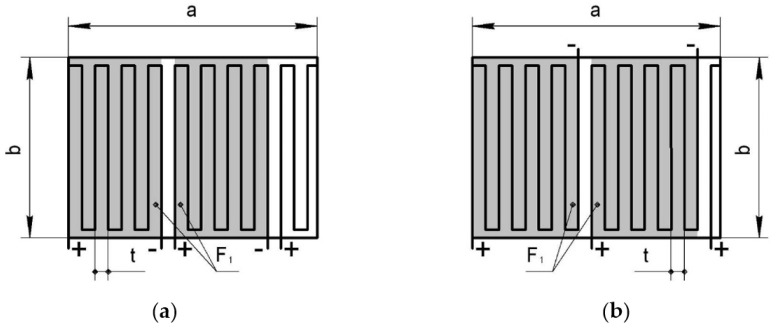
Patterns of laying for internal current sources (**a**) even number of pitches, (**b**) odd number of pitches.

It should be noted that for the pattern of laying of the elements of the fine-fibered resistive structure (Figure 4a), the *n* number has to be even, and for the pattern shown in Figure 4b it is an odd number.

Since we know the element laying area, the required heating area is represented as follows:

(14) $$F=mF_{1}+bt\left(m-1\right),$$

where *m*—coverage coefficient or coefficient of possible parallel connections, equal to:

(15) $$m=\frac{F+bt}{F_{1}+bt}=\frac{a+t}{nt}.$$

If *m* < 1, the heating wire material should be replaced, as shortening of the conductor can lead to a short circuit. At *m* ≥ 1 it is more advantageous to switch to the pattern of parallel conductor laying. The coverage coefficient is often a fractional number, which needs to be rounded down only. However, when changing the value of the coefficient, it is necessary to recalculate the conductor length, the laying pitch, the resistance, and the current strength.

Thus, the developed thermodynamic model allows determination of the time dependence of the specific power of internal heat sources in the resistive layer, which is necessary to maintain the given temperature regime on the middle surface of the moulded product. The constructed model takes into account the convective heat transfer from the outer surfaces of the heating system and the exothermic effect of the polymerization reaction. Further, according to the maximum value of the specific power, it is possible to determine the resistive element parameters in the heating system. 

## 4. Numerical Implementation

Numerical validation of the developed method was carried out on an example of moulding equipment for the formation of package based on fiberglass and Hysol EA 9396 binder, where the amount of heat Δ*H* = 400 kJ is released in the process of curing of one kilogram of the binder.

**Figure 5:**
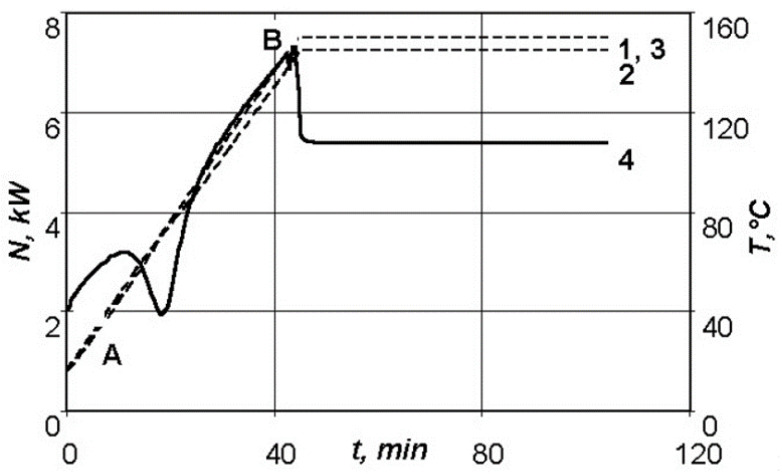
Dependences of parameters of the standard conditions of moulding of a package of 2 mm thick: 1—temperature versus time on the lower surface of the product; 2—temperature versus time on the upper surface of the product; 3—temperature versus time for the theoretical conditions; 4—power versus time.

Figure 5 shows the dependence of the required power of the resistive layer on the time required for maintenance of the given temperature–time regime on the lower surface of a moulded package of 2 mm thickness, as well as the distribution of the temperatures on the outer surfaces of the manufactured package of the polymeric composite material.

As can be seen in Figure 5, the sharp drop in the required power in section AB is explained by the exothermic effect of the curing reaction of the Hysol EA 9396 binder. As a result of self-heating of the system, the required power of the resistive layer noticeably drops in the time interval from 10 to 25 min, after which the graph of dependency of the required power on time becomes linear. The jump at point B is explained by the transition from the heating stage to the holding stage, at which the supplied energy is consumed only for the compensation of the convective heat transfer from outer surfaces of the thermodynamic system. An insignificant jump in temperature on the upper surface of the moulded package corresponding to the area of the required power dropping is explained by the fact that the presented thermodynamic system allows control of the temperature on one surface of the moulded product only. As previously determined, the choice of the lower surface as the control will prevent overheating of the structure on the upper layers of the moulded package, so that a higher quality product can be obtained.

**Figure 6:**
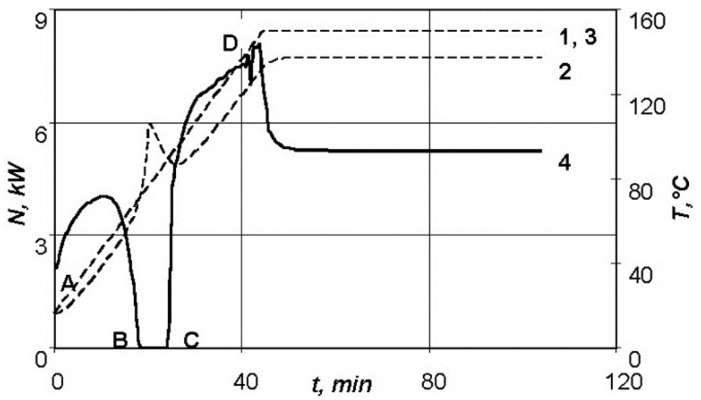
Dependences of parameters of the standard conditions of moulding of a package of 5 mm thick: 1—temperature versus time on the lower surface of the product; 2—temperature versus time on the upper surface of the product; 3—temperature versus time for the theoretical conditions; 4—power versus time.

Figure 6 shows the temperature–time relationship on the outer surfaces of the moulded product for a similar thermodynamic system, when the thickness of the moulded package is 5 mm, as well as the dependence of the required power of the heating system on time. In this case, the power of the resistive layer drops to zero at point B, which is explained by the growing exothermic effect of the curing reaction with the increase in thickness of the moulded package. Figure 6 shows that in section BC the exothermic effect of the curing reaction cannot be compensated only by convective heat removal from the outer surfaces of the system, and leads to the temperature peak on the lower surface of the moulded product at the heating section.

Therefore, the specified temperature–time regime for a product with such a thickness cannot be implemented using this equipment. Since large temperature gradients can lead to significant technological stresses, it is advisable to revise the conditions of moulding of the manufactured part, and possibly replace the binder used by another one, without such a pronounced exothermic effect.

The values of the required power, as well as the energy consumption for different heating rates of the thermodynamic system are presented in Table 1. It can be seen that the heat effect of the reaction growswith the increase in the heating rate of the product, and at a heating rate of 8 °C/min the power of the resistive layer drops to zero. Thus, variation of the rate of temperature rise in the moulded product is an effective way to control the power of the polymerization reaction exothermic effect. 

The material of the moulding tool also has a significant effect on the amount of consumed energy and the nature of its consumption. The values of the required power and energy consumption for various materials of the shaping surface of the tool when its thickness is changed are given in Table 2.

For example, for the fiberglass moulding tool, the power value gradually increases, excluding the area which balances the exothermic effect, while the metal tool is characterized by initially high energy consumption, associated with a high heat capacity ratio of the shaping surface material. The supplied heat is instantly redistributed inside the metal shaping surface and transferred to the environment due to convective heat removal. Within the composite shaping surface of the tool, heat is not distributed so quickly because of the complexity of the composite structure and its specific insulating properties; therefore, heat supply to the moulded package is faster than convective heat removal from the other surface of the moulding tool. 

**Figure 7:**
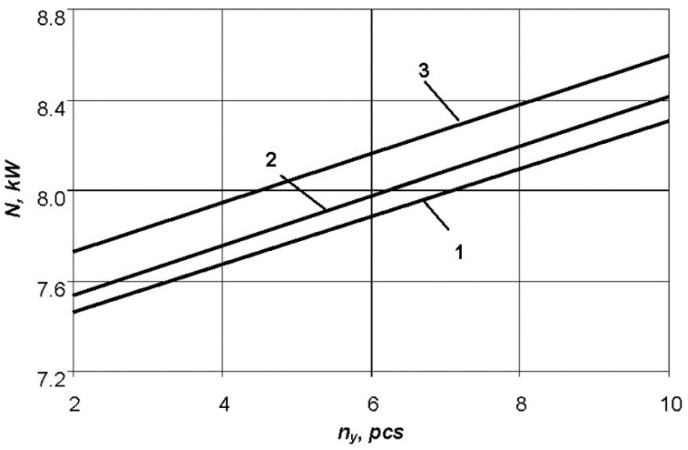
Graph of dependence of the power consumption on the number of stiffening ribs of various materials of the shaping surface of the tool: 1—fiberglass; 2—aluminium alloy; 3—steel.

The material and number of stiffening ribs of the moulding tool (*n_y_*) are important as well, as illustrated in Figure 7 where the highest value of heat removal is provided by steel ribs, which have maximum rigidity compared to the ribs made of aluminium alloy and fiberglass with the same dimensions of the ribs.

**Figure 8:**
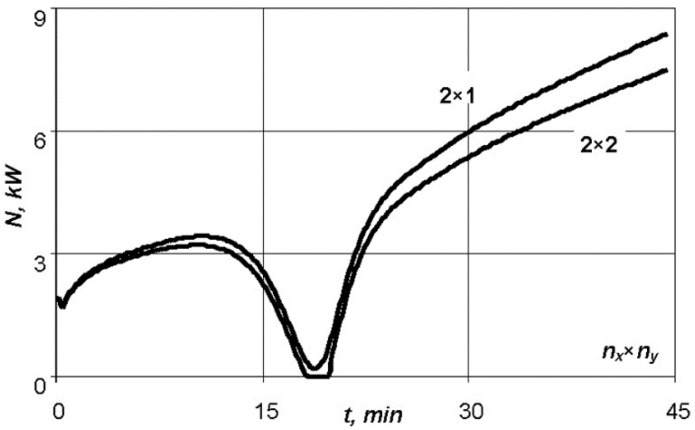
Graph of dependence of the power consumption on time for various numbers of aluminium stiffening ribs.

Besides, the number of ribs changes the conditions for heat exchange with the surface. So, for a small number of aluminium ribs it is not possible to maintain the specified temperature–time regime for a fiberglass moulded package of 5 mm thick with the use of this equipment (Figure 8). However, when the number of stiffeners is increased to 10, the exothermic effect of the reaction is smoothed and the heating equipment can then cope with the task.

## 5. Experimental Research

The moulding tool based on fiberglass and epoxy binder with the resistive element made of carbon thread was made for the moulding of test specimens with dimensions of 250 × 600 × 6 mm with the use of low-temperature binder. The tool met the following requirements: required moulding temperature—80 °C, resource—40 withdrawals, voltage from the power source—220 V.

According to the standard conditions of moulding for the furnace, the specimens were held for an hour at room temperature, after which they were heated at a rate of 1 °C/min to a temperature of 80 °C, and held for one hour. The system was cooled by heat exchange between the furnace and air.

**Figure 9:**
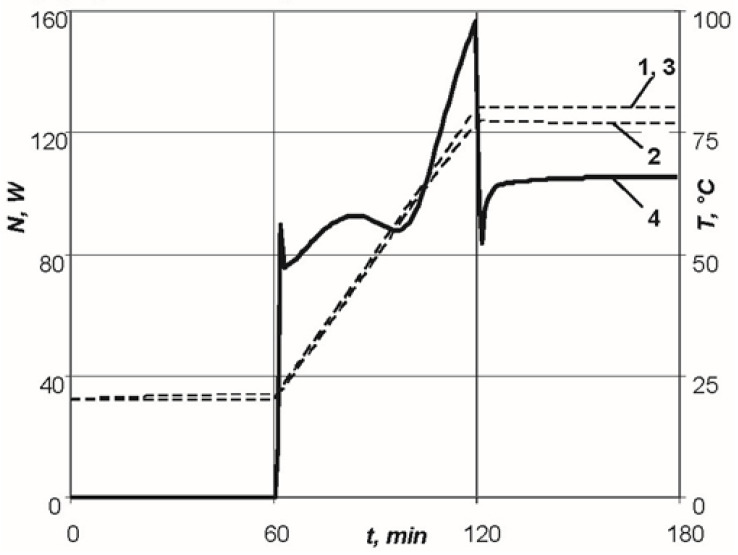
Dependences of parameters of the standard conditions of moulding for the package of 3 mm thick: 1—temperature versus time on the lower surface of the product; 2—temperature versus time on the upper surface of the product; 3—temperature versus time for the standard conditions; 4—power versus time.

To implement the specified moulding conditions, the heat conduction problem described previously was solved, and the maximum power of the resistive layer N= 158 W required for heating and temperature distribution on the outer surfaces of the moulded package were determined (Figure 9).

During holding at room temperature, spontaneous self-heating of the binder occurred, which led to 5 °C deviation from room temperature. During the second exothermic holding, the temperature value on the upper surface of the moulded package slightly increased as well. However, after 130 min of moulding the exothermic effect ceased and no deviations in temperature distribution from the specified one were observed.

**Figure 10:**
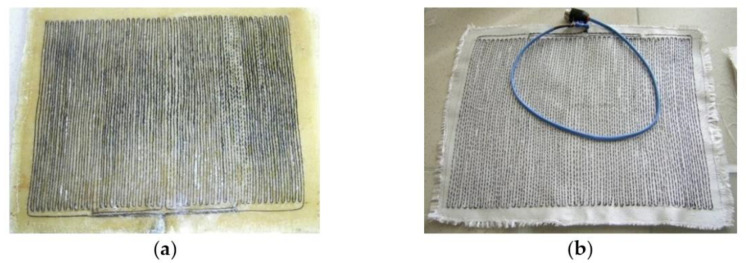
Heated moulding tool (**a**) and resistive layer based on carbon thread and fiberglass (**b**).

The shaping surface of the tool had a tolerance on perimeter (10 cm) to ensure easy withdrawal of the product and installation of the vacuum bag and fasteners along the contour. Thus, the dimensions of the shaping surface of the tool were 350 × 700 × 8 mm. The frame contained two stiffening elements (of rectangular section, of 20 × 10 mm in size) installed in the transverse direction of the larger side (Figure 10).

**Figure 11:**
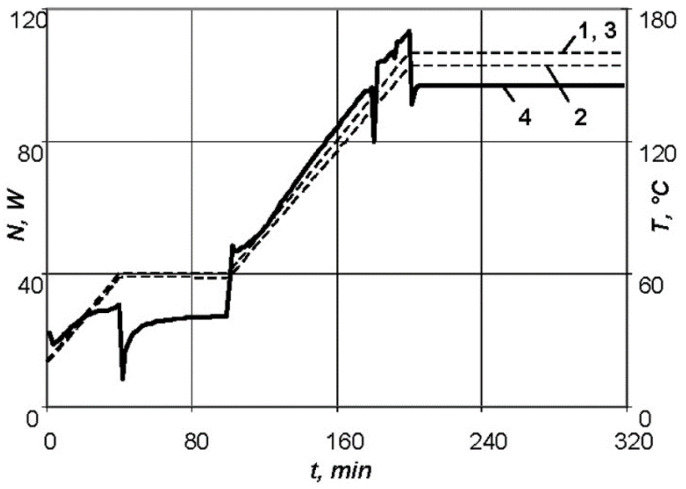
Dependences of parameters of the standard conditions of moulding for a patch of 2 mm thick: 1—temperature versus time on the lower surface of the product; 2—temperature versus time on the upper surface of the product; 3—temperature versus time for the standard conditions; 4—power versus time.

**Figure 12:**
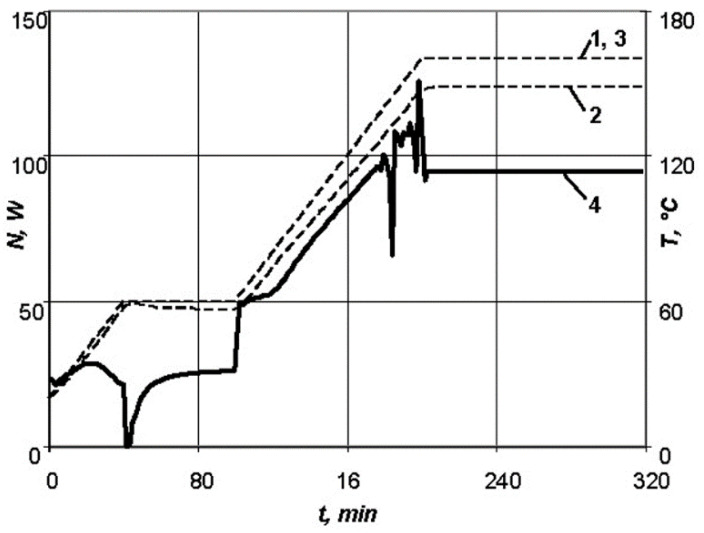
This is a figure. Schemes follow the same formatting. Dependences of parameters of the standard conditions of moulding for a patch of 4 mm thick: 1—temperature versus time on the lower surface of the product; 2—temperature versus time on the upper surface of the product; 3—temperature versus time for the standard conditions; 4—power versus time.

The necessary characteristics of the resistive layer were determined. Carbon fibre with a radius of 0.315 mm, maximum breakdown current of 0.3 A, and resistance per unit length of 500 Ohm/m was used as the heating wire of the resistive layer. The required length of the heating wire equal to 1.76 m was calculated for the series connection. The carbon thread laid in serpentine rows with a pitch of 6 mm, was sewn onto a layer of fiberglass and formed four parallel connections. The resistive layer was moulded into the shaping surface at a depth of 3 mm. The heating system power was equal to 220 W. A flexible thermal blanket was made for the repair of non-separable metal and composite structures in case of appearance of small cracks or other defects during operation. The repair was carried out by patching of the skin under repair, followed by heating of the thermal blanket, which takes any shape of the structure being repaired. Requirements for the thermal blanket: size of 225 × 225 × 6 mm, were made on the basis of high-temperature silicone and carbon thread, maximum temperature—160 °C, voltage from power source—220 V. To implement the standard conditions of moulding [34], the following values were determined: the maximum power of the resistive layer required for heating of an aluminium skin of 2 mm thickness under repair and a fiberglass patch of 2 mm thick—*N* = 113 W (Figure 11), as well as for a patch of 4 mm thick—*N* = 129 W (Figure 12); the temperature distribution over surfaces of the composite patch.

At the stage of heating up to the first exothermic holding, the exothermic effect grows in the product, but is easily controlled by changing the supplied power of the heating equipment when moulding a patch of 2 mm thick. However, for a patch thickness of 4 mm it is not possible to smooth the exothermic effect.

Depending on the thickness of the panel under repair, the value of the required power of the heating system changes; some values of which are presented in Table 3.

**Figure 13:**
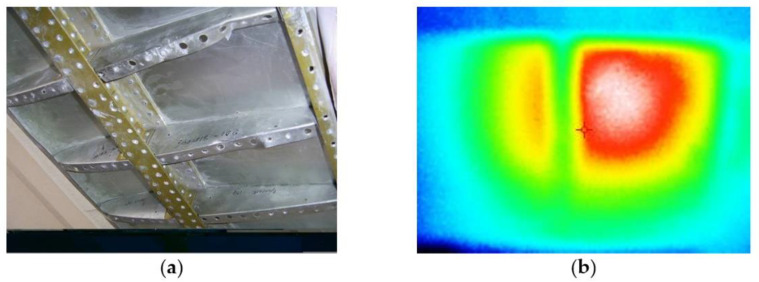
Panel under repair (**a**) and heat loss on its rib (**b**).

In addition, the presence of ribs on the repaired panel and their number and layout can qualitatively change the pattern of temperature distribution over the surface (Figure 13). For a panel under repair of 2 mm thick, with the parameters of the stiffening ribs of 20 × 10 mm, the change in the amount of energy consumed is presented in Table 4.

**Figure 14:**
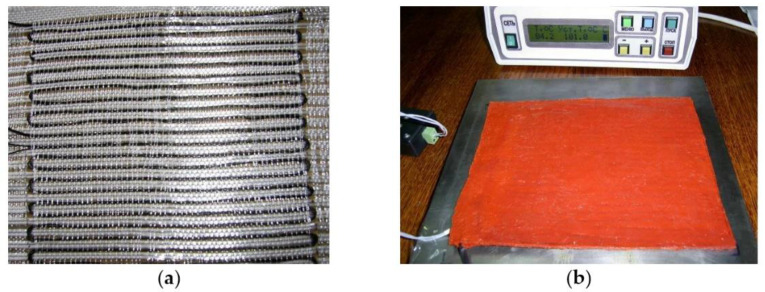
Resistive layer based on carbon thread and fiberglass (**a**) and flexible thermal blanket (**b**).

Carbon fibre with a radius of 0.0315 cm, maximum breakdown current of 0.3 A, and resistance per unit length of 500 Ohm/m was used as the heating wire. The required length of the heating wire for the series connection was equal to 1.76. The resistive layer (Figure 14) was formed on the basis of four resistive blocks connected in parallel, which were laid in two. The resistive structure was drawn through the layer of fiberglass in serpentine rows of 6 mm pitch, with two strands in a hemstitch. High-temperature silicone was used as a nonconductive layer, deposited on both sides of the fiberglass with an extended resistive structure. The thickness of the thermal blanket was 6 mm, which ensured a uniform temperature distribution over the surface. The power of the heating system (220 W) was sufficient for moulding of patches of 2−4 mm thick.

## 6. Discussion

A method for determination of the parameters of the moulding tool resistive layer was developed. This method allows the heating layer parameters to be calculated and the specified time–temperature regime for moulding of the composite structure to be implemented. Numerical research allowed evaluation of the effectiveness of this moulding method [31,34] compared to the traditional one [24,25]. During the moulding of experimental fiberglass specimens, energy saving with the use of the shape-generating moulding tools with internal heating is between 40 and 60%, depending on the adaptability of the moulding parameters to the equipment, which provides the heating (Table 5) [25,34]

A thermodynamic model of unsteady heat transfer for the “surface shaping-polymer composite material” system was developed [34]. The model in contrast to that in other papers [28,29] allows the temperature distribution over the thickness of the system to be obtained, the influence of the exothermic effect of the binder curing reaction to be evaluated, and the required power of the heating system to be determined. Furthermore, the model in contrast to paper [34] considers the ribbing of the lower surface of the moulding tools.

It was found that the increase in the thickness of the moulded package of the polymeric composite material resulted not only in a higher supplied power of the heating system, but also in a complication of the method for system control, because of the growing exothermic effect of the binder curing reaction. The results obtained partly confirm the preliminary conclusions from other works [29,31,32].

The influence of the physical and mechanical characteristics of the moulding tool material and stiffening ribs was analysed in terms of energy consumption and controllability of the heating system similar to the papers [33,34]. Fiberglass shows the lowest energy consumption. Heating of the aluminium and steel moulding tools for the same purpose will require 20% and 45% more power, respectively. The results obtained partly confirm the preliminary conclusions from other works [24,25,27].

In contrast to the papers [32,34], the increase in the number of stiffening ribs has a strong effect on the heat removal of the heating system. For binders with an unpronounced exothermic effect, the presence of the frame in the structure of the moulding tool leads to an increase in the energy consumption of the system. However, for binders with a strongly pronounced exothermic effect, the presence of the frame allows the controllability of the heating process to be improved and the given time—temperature regime to be achieved when the system is being cooled with free convection only. For the first time, the dependences of the parameters of the heating system on the heating rate are shown. As the heating rate grows, the power consumption increases, but the energy consumption of the system decreases. In this case, the controllability of the heating system is reduced, since the self-heating reaction of the binder increases with the implementation of high heating rates. 

An experimental prototype of heating equipment for a moulding tool for the manufacture of structures of polymeric composite materials, as well as a flexible thermal blanket for the repair of non-separable metal and composite structures, were developed.

The obtained results in the end make it possible to reduce the energy consumption for internally heated moulding tools by choosing the optimal parameters for the resistive layer.

## 7. Conclusions and Further Research

A method for determination of parameters of the resistive layer of moulding tools was developed, which allowed calculation of the heating layer parameters and implementation of the specified time–temperature regime for moulding of composite structures.

A thermodynamic model of unsteady heat transfer for the system “shaping surface-polymeric composite material” was constructed.

An experimental prototype of heating equipment for the moulding tools for the manufacture of structures of polymeric composite materials was developed.

The results of the study can be the basis for a new method of optimal design of parameters of moulding tool structure at minimal heat removal to the environment.

## Figures and Tables

**Table 1:** Required power and energy consumption values for different heating rates.

Heating Rate, °C/min	Required Power of Resistive Layer, kW	Energy Consumption, kW/h
1	6.15	7.16
2	6.78	4.17
3	7.51	3.18
4	8.10	2.68
5	8.78	2.38
6	9.46	2.19
7	10.15	2.04
8	10.83	1.94

**Table 2:** Required power and energy consumption values for the heating system of moulding tool when the thickness of lower part of the shaping surface is changed.

Thickness of Lower Part of the Shaping Surface, mm	Required Power of the Heating System, kW	Energy Consumption, kW/h	Required Power of the Heating System, kW	Energy Consumption, kW/h	Required Power of the Heating System, kW	Energy Consumption, kW/h
	Fiberglass		Aluminium Alloy		Steel	
0.5	6.99	2.81	7.06	2.83	7.20	2.90
1	7.16	2.94	7.37	2.97	7.46	3.12
2	7.51	3.18	7.71	3.27	8.16	3.57
4	8.08	3.62	8.49	3.86	9.91	4.45
6	8.56	4.01	9.44	4.45	10.53	5.34
8	8.96	4.33	10.35	5.04	12.18	6.22
10	9.44	4.60	11.44	5.62	12.87	7.10

**Table 3:** Change in the power of the heating system when the thickness of the panel under repair is changed.

Metal Panel Thickness, mm	2	4	6	8
Required power, W	113	122	127	142

**Table 4:** Change in the power of the heating system when the number of stiffening ribs is changed.

Number of Ribs n_x_ × n_y_, pcs.	2 × 2	2 × 3	2 × 4	3 × 4
Required power, W	113	153	164	169

**Table 5:** Comparison of energy consumption of traditional and new equipment.

Parameters of Specimen Curing Mode	Electric Furnace		Moulding Tool with Internal Heating		∆*N*,%
	*τ*, h	*N_n_*, W	*τ*, h	*N_m_*, W	
*V_1_* = 1 °C/min, *V_2_* = 1 °C/min, *T_1_* = 60 °C, *T_2_* = 160 °C, *t*_1_ = 60 min, *t_2_* = 120 min	14.3	940	5.63	587	40
*V_1_* = 1 °C/ min, *V_2_* = 1 °C/min, *T_1_* = 60 °C, *T_2_* = 160 °C, *t_1_* = 60 min, *t_2_* = 65 min	12.6	67	4.13	250	63
